# MicroRNA and Transcription Factor Gene Regulatory Network Analysis Reveals Key Regulatory Elements Associated with Prostate Cancer Progression

**DOI:** 10.1371/journal.pone.0168760

**Published:** 2016-12-22

**Authors:** Mehdi Sadeghi, Bijan Ranjbar, Mohamad Reza Ganjalikhany, Faiz M. Khan, Ulf Schmitz, Olaf Wolkenhauer, Shailendra K. Gupta

**Affiliations:** 1 Department of Biophysics, Faculty of Biological Sciences, Tarbiat Modares University, Tehran, Iran; 2 Department of Biology, Faculty of sciences, University of Isfahan, Isfahan, Iran; 3 Department of Systems Biology and Bioinformatics, University of Rostock, Rostock, Germany; 4 Gene and Stem Cell Therapy Program, Centenary Institute, University of Sydney, Camperdown, Australia; 5 Sydney Medical School, University of Sydney, Camperdown, Australia; 6 Stellenbosch Institute for Advanced Study (STIAS), Wallenberg Research Centre at Stellenbosch University, Stellenbosch, South Africa; 7 Department of Bioinformatics, CSIR-Indian Institute of Toxicology Research, Lucknow, India; Qom University, ISLAMIC REPUBLIC OF IRAN

## Abstract

Technological and methodological advances in multi-omics data generation and integration approaches help elucidate genetic features of complex biological traits and diseases such as prostate cancer. Due to its heterogeneity, the identification of key functional components involved in the regulation and progression of prostate cancer is a methodological challenge. In this study, we identified key regulatory interactions responsible for primary to metastasis transitions in prostate cancer using network inference approaches by integrating patient derived transcriptomic and miRomics data into gene/miRNA/transcription factor regulatory networks. One such network was derived for each of the clinical states of prostate cancer based on differentially expressed and significantly correlated gene, miRNA and TF pairs from the patient data. We identified key elements of each network using a network analysis approach and validated our results using patient survival analysis. We observed that *HOXD10*, *BCL2* and *PGR* are the most important factors affected in primary prostate samples, whereas, in the metastatic state, *STAT3*, *JUN* and *JUNB* are playing a central role. Benefiting integrative networks our analysis suggests that some of these molecules were targeted by several overexpressed miRNAs which may have a major effect on the dysregulation of these molecules. For example, in the metastatic tumors five miRNAs (*miR-671-5p*, *miR-665*, *miR-663*, *miR-512-3p* and *miR-371-5p*) are mainly responsible for the dysregulation of *STAT3* and hence can provide an opportunity for early detection of metastasis and development of alternative therapeutic approaches. Our findings deliver new details on key functional components in prostate cancer progression and provide opportunities for the development of alternative therapeutic approaches.

## Introduction

Prostate cancer is the second leading cause of cancer death after lung cancer in the United States [1]. The number of latent or undetected prostate cancer incidences even outnumbers those that were clinically detected. The intense heterogeneity in prostate cancer is an outcome of genetic variations underlying this disease. There are a number of genes involved in prostate cancer progression, which have been reported to be downregulated in some studies and overexpressed in other studies (e.g. *STAT3*) [2,3]. A large number of studies are based on a reductionist approach to confirm the role of one or another gene or signaling pathway as a key player in prostate cancer metastasis [4–8]. However, this approach fails to describe the mechanisms of tumor progression and metastasis and the link to clinical phenotypes. Phenotypic outcomes of the disease can however be studied using a systems biology approach by modeling the impact of signaling cascade dysregulation on metastasis [9,10].

Technological advances in high throughput data generation from multiple system levels provide a comprehensive view on different biological phenotypes. For example, there is a very close regulatory relationship between gene expression and miRNA expression that can be studied using current high-throughput transcriptomics techniques. MiRNAs are a class of small (22 nucleotide), non-coding RNAs that regulate gene expression in a sequence-specific manner and are involved in almost all cellular processes. MiRNAs are able to regulate gene expression at post-transcriptional level by binding to partially complementary mRNA sequences and mediating degradation of that mRNA. MiRNA dysregulation is often involved in cancer progression as a cause for tumor suppressor gene repression or oncogene de-repression, both leading to disease phenotypes [11,12].

Many TFs are already known to play a crucial role in the regulation of prostate cancer metastases. For example, *STAT3*, *AP1*, *AR*, *ERG*, *EGR1* and *MYC* are important TFs that are responsible for the progression of prostate cancer by affecting a wide variety of pathways involved in cell proliferation and differentiation, apoptosis, tumor suppressing and various cell signaling pathway [13–16]. Available information about TFs, their targets and associated pathways is increasing. Therefore, data on TF abundance in a given phenotype allows us to infer the effect on target genes and pathways and their role in the regulation of the phenotype. Gene expression regulation is an intricate process, which is controlled by diverse factors including proteins and RNAs.

TFs and miRNAs are two key regulators that control (directly or indirectly) their own expression and the expression of their mutual targets in the form of feedback and feed-forward loops [17,18]. TF and miRNA co-regulation is prevalent in biological systems and any perturbation to this co-regulation can lead to a malfunctioning system and disease [19]. Integrating gene expression, TF regulatory network and miRNA regulatory network analyses can help surveying key genomic factors and, importantly, also their interactions, which may provide insights into the causes of disease. This also unravels interrelationships between pathways that are involved in the disease progression. Combining multiple data types can compensate for missing or unreliable information in any single data type as multiple sources of evidence pointing to the same gene or pathway are less likely to lead to false positive results which is very important in complicated pathologies like prostate cancer [20].

In our study we used data from 218 prostate tumor samples to generate an integrated regulatory network for differentially expressed biological entities (miRNAs, mRNAs and TFs) in primary and metastatic prostate cancer phenotypes utilizing the data by Taylor and colleagues in [21]. The network construction was restricted to only significantly correlated gene-TF-miRNA pairs. We further analyzed our differentially expressed networks based on various topological parameters in order to identify important trios, i.e. a loop composed of mRNA, miRNA and TF, which can distinguish primary vs. metastatic stages.

Our analysis resulted in a small set of regulatory signatures, which we validated using survival analysis in patient data. These signatures may be considered in the design of novel therapeutic approaches or as potential biomarkers.

## Methods

### Data sets

The microarray data used in the present study was retrieved from the Gene Expression Omnibus (GEO, GSE21032). The gene expression profiles were measured with the Affymetrix Human Exon 1.0 ST Array platform, and image quantification was performed using the GeneChip Operating Software (GCOS) version 1.4. The miRNA expression profiles were measured on the Agilent-019118 Human miRNA Microarray 2.0 G4470B platform and the images were quantified using Agilent Feature Extraction version 9.5.

### Microarray data preprocessing and normalization

Statistical analyses and calculations were performed using R, version 3.2.2. Human exon array preprocessing (background correction, normalization and summarization) were performed using the aroma.affymetrix R package. The RMA method was implemented for gene expression data normalization. Core probe sets, i.e. the probe sets that are supported by the most reliable evidence from RefSeq and full-length mRNA GenBank records containing complete CDS information, were used for further analysis. Normalized expression data was subjected to log_2_ transformation for further analysis. Differential gene expression analyses for genes and miRNAs were performed using linear regression models in the limma R package [22]. The limma package uses *t-statistics* for comparing each gene expression value in each comparison groups. Genes and miRNAs with an absolute log fold change greater than 1 and p-value less than 0.05 were selected as differentially expressed. Benjamini-Hochberg (BH) multiple testing correction was applied on the *t-statistics* analysis results.

### Functional annotation

The Cytoscape plugin BiNGO was applied to detect significantly overrepresented GO biological processes and graphical representation of these terms [23].

### TF and miRNA regulatory analysis

The TRANSFAC database was used for finding TFs, their experimentally validated target genes among the differentially expressed genes (DEGs), and their binding site distribution in the target promoter regions. Pearson’s correlation coefficient (PCC) for expression value and corresponding p-value for each TF and target gene pairs were calculated. Pairs with absolute PCC value more than 0.4 and p-value less than 0.05 were selected as significant TF and target gene pair. The binding site (BS) distribution of key TFs in both primary and metastatic prostate cancer networks have been identified using the information available on TF binding sites in the TRANSFAC database. We compared the BS of key TFs with random gene sets selected from the networks. For this 10% of DEGs other than those with good Pearson’s correlation with the key TFs in the primary and metastatic prostate tumors were selected as random sets. Furthermore, we used MirTarbase [24], miRanda [25] and TargetScan [26] to identify target genes among DEGs specifically for differentially expressed miRNAs (DEMs) in the patient data. The pairs of miRNAs and genes with absolute PCC values higher than 0.4 were considered for the selection of important regulatory signatures. Finally, regulatory networks were constructed for primary and metastatic prostate tumors by merging selected DEMs-DEGs and TFs-DEGs pairs using Cytoscape.

### Network parameter analysis

We used the NetworkAnalyzer plugin in Cytoscape for the calculation of betweenness centrality and node degrees [27].

### Kaplan-Meier survival analysis

Biochemical recurrence (BCR) free survival probability curves for each group were calculated using Kaplan-Meier survival curves and the significance of difference between each group pair analyzed by the log-rank test. We performed Kaplan-Meier survival analysis in R using Survival package [28].

## Results

For our integrated regulatory network analysis in prostate cancer, we retrieved patient-derived data from Gene Expression Omnibus. More specifically we used the data generated in Taylor et al., who conducted a comprehensive genomic analysis on 218 prostate tumor samples, which include samples from the primary and metastatic stages of the disease [21]. From the 218 biological samples in the super-series, we found 139 samples with both mRNA and miRNA expression profiles among which 98 samples were taken from primary tumors, 13 from metastatic tumors and 28 from normal prostate tissue. The processed miRNA expression data contains expression profiles of 375 unique miRNAs.

### Differential expression analysis

**Fig 1:**
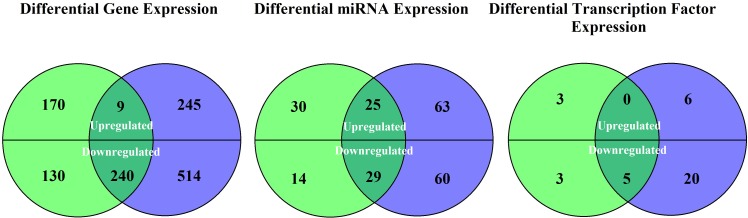
Venn diagrams of differentially expressed regulatory components in prostate tumor. The number of DEGs, DEMs and TFs in primary and metastatic tumor samples (green and purple ovals, respectively) are shown. It is interesting to note that primary and metastatic tumors do not share any upregulated TFs.

For finding DEGs and DEMs (1) primary prostate tumor samples were compared to normal prostate tissue samples and (2) metastases prostate cancer samples were compared to primary prostate cancer samples. We found 549 genes in primary tumors and 1008 genes in metastasis to be differentially expressed in the study (p-value < 0.05 and log fold change (LFC) > 1). From these, 179 genes were upregulated in the primary state and 254 genes in the metastatic state, while 370 genes were downregulated in the primary state and 754 genes in the metastatic state (Fig 1).

**Fig 2:**
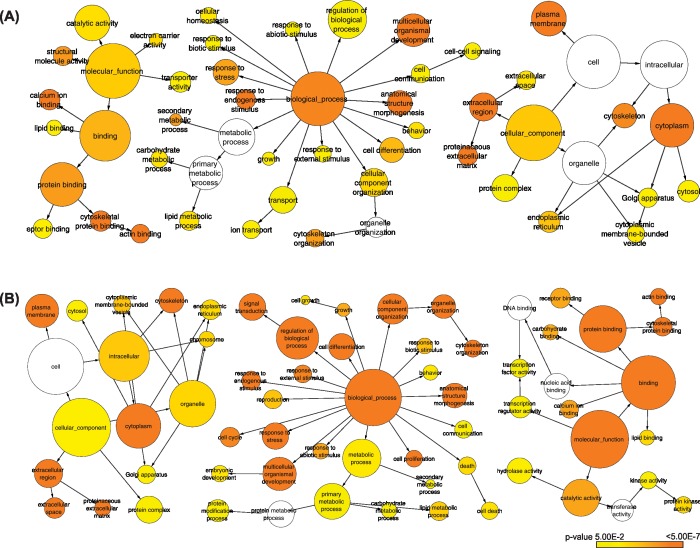
**GO term enrichment analysis for differentially expressed genes in (A) primary and (B) metastatic prostate tumor samples.** The nodes color represents the significance of the GO terms and the node size represents number of genes in that category.

As disease progresses to the metastatic state more genes and more biological processes and pathways are dysregulated in the system (Fig 2). In order to detect biological processes affected by these changes we performed a functional enrichment analysis of the DEGs. Significantly overrepresented GO biological processes for primary tumors include cell differentiation, cell communication and growth. For the metastatic state pathways involved in cell proliferation, cell cycle, cell differentiation, cell death, extracellular region, and signal transduction, specifically the ones involved in response to cytokines were enriched.

In the miRNA expression data we found 98 and 177 miRNAs differentially expressed in primary and metastatic states of prostate cancer respectively (p-value < 0.05 and LFC > 1). In the primary tumor state 55 miRNAs were upregulated and 43 miRNAs downregulated, whereas in the metastatic state 88 miRNAs were upregulated and 89 miRNAs downregulated (Fig 1). We selected these DEMs for further analysis.

### TF regulatory network analysis

**Fig 3:**
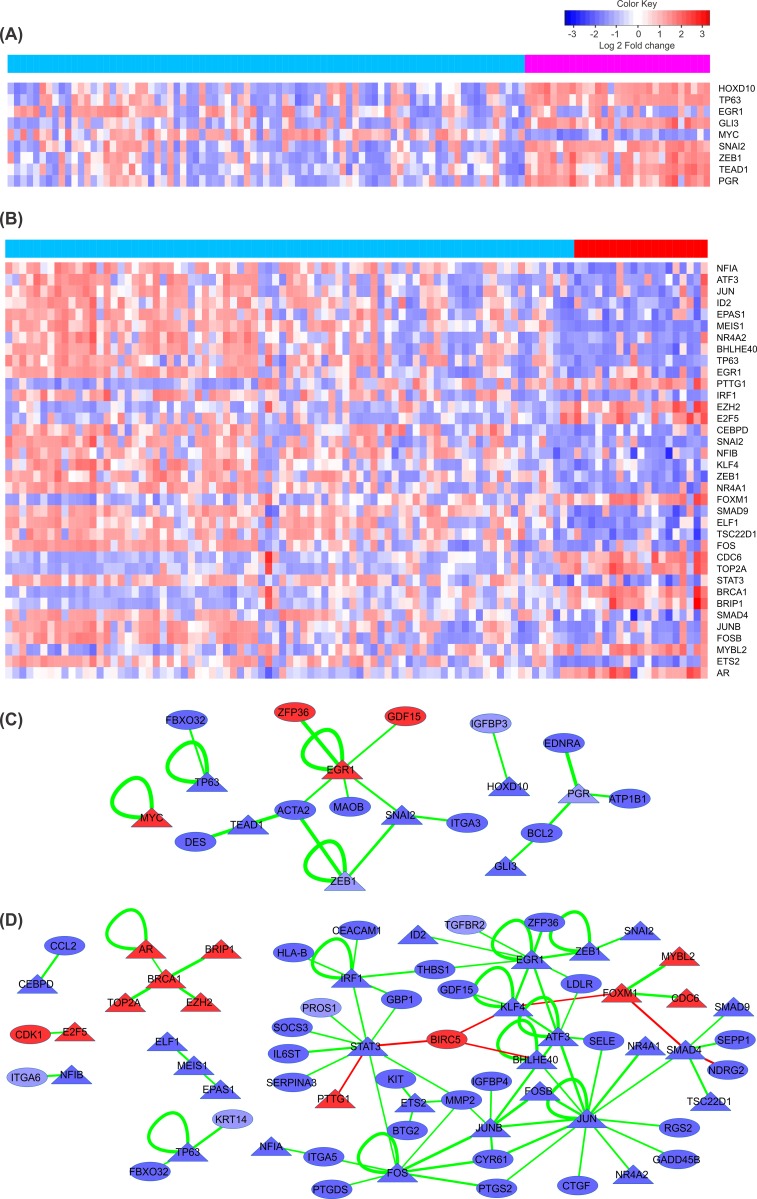
Expression patterns of differentially expressed TFs and TF regulatory networks for primary and metastatic prostate cancer. Heat map of differentially expressed TFs having significant correlations with differentially expressed target genes in primary (A) and metastatic tumors (B). Each row represents a TF and each column represents a different sample. Color bars above the columns represent groups of samples: light blue, red and magenta for primary, metastatic and normal samples, respectively. Cells represent z-scores of TF expression values ranging from blue for low expression to red for highly expressed TFs. TF regulatory interactions with corresponding genes have been represented for primary (C) and metastatic (D) tumors. TFs and target genes are shown as triangular and oval nodes. The node color represent log fold changes (blue: down-regulation; red: up-regulation). The edge color indicates the type of regulation (green for activation and red for repression) and the edge width is proportional to the absolute correlation coefficient for the expression values of the connected pair.

549 DEGs in primary tumors and 1008 DEGs in metastases were used to build a regulatory network of differentially expressed TFs (DETF) and corresponding target genes. The TRANSFAC database, which is a comprehensive and unique knowledge-base containing published data on eukaryotic TFs and miRNAs, their experimentally-proven binding sites, and regulated genes, was used to detect DETFs and their targets among differentially expressed genes [29]. The PCC of gene expression values was used to identify potential regulatory pairs of DETFs and DEG targets. We found 23 significant pairs of DETFs and target genes (absolute PCC > 0.4) in the primary state of the disease, which involve in total 10 TFs and 18 target genes. In case of metastatic stage 60 significant pairs (absolute PCC > 0.4) were observed involving 20 unique DETFs and 48 corresponding target genes (Fig 3) (S1 and S2 Tables).

### MiRNA regulatory network analysis

MiRNAs have a significant impact on the regulation of the key molecular signatures in integrative regulatory networks [30]. In many cases, miRNAs regulate TFs and target genes through different regulatory circuits including feedback and feed-forward loops. For example, Vera *et al*. described an incoherent feed-forward loop, composed of E2F1, p73, DNp73 and miRNA-205, regulating melanoma progression [31].

**Fig 4:**
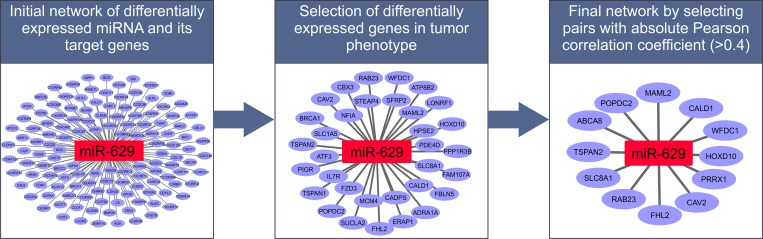
Workflow for the identification of functional miRNA-mRNA interactions. The workflow indicates the selection of small set of disease and phenotype specific functional targets from a large number of potential target genes for *miR-629*. In the first step *miR-629* is connected with targets derived from databases of experimentally validated and predicted target genes (left). In the second step only those targets are preserved that are differentially expressed in prostate cancer (middle). Finally, functionally relevant *miR-629* targets are determined based on a significant correlation between *miR-629* and target gene expression (right).

98 DEMs in primary tumors and 179 DEMs in metastases, which satisfy our criteria, (p-value < 0.05 and LFC > 1) defined in the previous step, were selected for further analysis. We integrated data on experimentally validated and predicted miRNA target genes for identifying targets of DEMs. There should be a negative correlation between miRNAs and their corresponding target genes because miRNAs suppress target gene expression. However, in certain situations mRNA levels of miRNA targets do not change as is the case in the mechanism of translation suppression where only target protein levels are affected by miRNA regulation [32]. Even positively correlating miRNA and mRNA expression profiles are sometimes being observed as in [33], which is in most of the cases a consequence of indirect target regulation, for instance, when a miRNA targets a transcriptional suppressor of another gene. For these reasons, we identified all those DEM-DEG pairs where the absolute PCC value was greater than 0.4. In our analysis we considered only those genes as potential targets of DEMs that were among the list of DEGs. We found 1664 potential regulatory interactions from 57 unique DEMs and 409 unique DEGs in primary state; and 5288 potential regulatory interactions from 96 unique DEMs and 842 unique DEGs in metastatic state which we derived from databases of experimentally validated and predicted miRNA target genes. Correlation analysis on these potential regulatory pairs reveals that 363 regulatory interactions between 41 miRNAs and 190 genes in primary state (280 negatively correlating and 83 positively correlating regulatory interaction) and 621 regulatory interactions between 79 miRNAs and 346 genes in metastatic state (490 negatively correlating and 131 positively correlating regulatory interaction) have significant correlations (absolute PCC > 0.4; S1 Fig). The efficiency of the implemented approach in narrowing down functional miRNA-target interactions is illustrated in Fig 4.

### Integrating TF regulatory networks and miRNA regulatory networks

**Fig 5:**
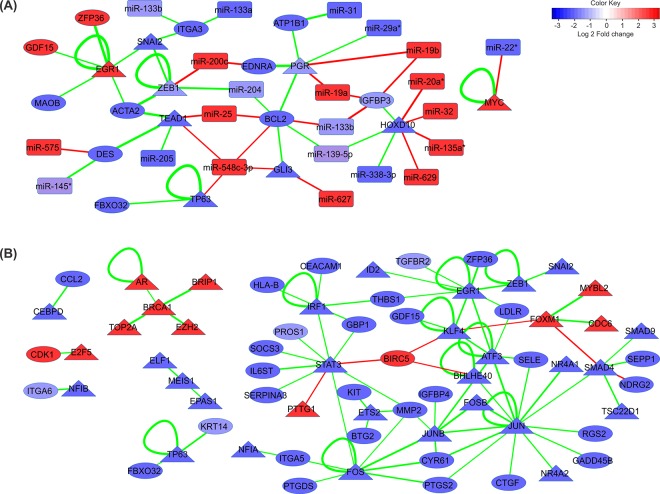
**Integrative miRNA and TF regulatory networks for (A) primary; and (B) metastatic prostate cancer**. MiRNA, genes and TFs are represented as rectangular, circular and triangular nodes respectively. Nodes are colored based on their log fold change (blue: down-regulation; red: up-regulation). The edge color indicates the type of regulation (green for activation and red for repression) and the edge width is proportional to the absolute correlation coefficient for the expression values of the connected pair.

For the construction of the interacted regulatory networks we extended the TF-DEGs network shown in Fig 3 with significantly correlating miRNAs where the absolute PCC value between the nodes in TF-gene regulatory network and miRNAs were higher than 0.4. The integrated regulatory networks were constructed and visualized for both clinical stages in prostate cancer progression using Cytoscape [34]. Our integrated networks contain 52 and 143 significant regulatory interactions in the primary and metastatic states respectively. In the primary state 9 DETFs, 11 DEGs and 21 DEMs are involved in the integrated network and in the metastatic state 36 DETFs, 32 DEGs and 34 DEMs are involved in the integrated network (Fig 5).

#### Network analysis

The number of elements involved in the primary integrative regulatory network is small in contrast to that of the metastases state. In order to identify key structural elements in the integrated miRNAs and TFs regulatory network, two important topological network parameters were considered: (i) the degree, and (ii) the betweenness centrality of each node in the network. These two parameters describe different characteristics of nodes in the network. The node degree is a local centrality measure that counts the number of other nodes a node is directly connected to. It highlights that an important node is involved in a large number of interactions. It is a measure of how well connected a node is in a network. Nodes with very high degree are called hubs since they are connected to many neighbors. The betweenness centrality of a node reflects the amount of control that a node exerts over the interactions of other nodes in the network. This measurement favors the nodes that act as connecting links between dense subnetworks, rather than nodes that lie inside a subnetwork [35]. More precisely, the betweenness centrality *BC*(ʋ) of a node *ʋ ϵ V* is the fraction of shortest paths between pairs of nodes u, w ϵ V that pass through ʋ:

$$BC(ʋ)=\sum_{u,w\epsilon V}\frac{\sigma_{uw}(ʋ)}{\sigma_{uw}},\;where\;u\neq w\neq ʋ$$

Where, σ_uw_(ʋ) denotes the total number of shortest paths between *u* and *w* that pass through node *ʋ* and σ_uw_ denotes the total number of shortest paths between *u* and *w*.

We considered nodes that have values 2 standard deviations higher than the average degree and betweenness centrality (z score > 2) as key vertices in the integrated miRNA/TF regulatory networks. Integrative regulatory network analysis revealed 3 key elements in both the primary and the metastatic prostate tumor networks (Table 1).

**Table 1:** Integrative regulatory network analysis results based on two network structure parameters (degree and betweenness centrality).

*Integrative gene regulatory network analysis results*			
	*Node ID*	*Degree*	*Betweenness Centrality*
***Primary***	*BCL2*	7	0.51
	*PGR*	6	0.26
	*HOXD10*	7	0.25
***Metastases***	*STAT3*	17	0.33
	*JUN*	15	0.28
	*JUNB*	8	0.22

Among these elements are two TFs (*PGR* and *HOXD10*) in the primary and three TFs (*STAT3*, *JUN* and *JUNB*) in the metastatic prostate cancer network. We calculated the number of binding sites for these key TFs in the promoter region of target genes present in the respective networks. Also, the binding site distribution of these TFs in the promoter regions of random gene sets has been identified. We compared these two frequencies and found that the binding site frequency in identified target genes of key transcription factors in the networks is significantly higher than those of the random gene sets (S2 Fig).

*HOXD10* previously was reported as downregulated in prostate cancer cells [36]. It encodes a nuclear protein which functions as a sequence-specific TF. It regulates multiple downstream genes including *IGFBP3* (also present in our network) which is a pro-apoptotic and anti-angiogenic protein in prostate cancer and activates caspase 3 and caspase 8, and subsequently induces cell apoptosis [37]. Low *IGFBP-3* levels are associated with greater risk of aggressive prostate cancers [8]. Based on the integrative regulatory network for primary state *HOXD10* and its target gene *IGFBP3* are targets of multiple overexpressed miRNAs *(miR-20a*, *miR-32*, *miR-135a* and *miR-629* for *HOXD10*, and *miR-19a*, *miR-19b* and *miR-375* for *IGFBP3*) which might be the reason for the suppression of the mentioned genes in the primary prostate tumor. *PGR* encodes the progesterone receptor, which belongs to the same steroid receptor family as *AR* and *ER*. Elevated levels of *PGR* suppress prostate stromal cell proliferation through the inhibition of cyclinA, cyclinB, and cdc25c, which consequently interrupts the cell cycling process [38]. On the other hand, an association of *PGR* expression with aggressive phenotypes of prostate cancer was also reported [39]. However, the role of *PGR* in the prostate cancer has not been fully understood yet. Regulation of *BCL2* by *PGR* through direct binding to its promoter has been reported before [40,41]. *BCL2* is an anti-apoptotic onco-protein. Increased expression of *BCL2* contributes to tumorigenicity, poor clinical prognosis and resistance to chemo- and radiation-therapy in many tumors [42,43]. Up-regulation of *BCL2* correlates with progressed levels of prostate cancer but the exact role of BCL2 in prostate cancer is not explicit. *BCL2* and *PGR* are targeted by overexpressed miRNAs in the integrative regulatory network (*miR-25*, *miR-375* and *miR-548c-3p* for *BCL2*, and *miR-19a* and *miR-19b* for *PGR*).

The majority of molecular signatures in the metastatic integrative regulatory network were downregulated (Fig 5B). *STAT3*, *JUN* and *JUNB* were identified as key signatures in the regulatory network analysis. Among these genes *STAT3* is the most interesting one. This gene is a member of the STAT (Signal Transducers and Activators of Transcription) family. It is implicated in programming gene expression in biological events such as programmed cell death, organogenesis, innate immunity, adaptive immunity, cell growth regulation and embryonic development, in many organisms [44]. *STAT3* acts as signal messenger and TF and participates in normal cellular responses to cytokines and growth factors specifically to *IL6*. *STAT3* overexpression is thought to be oncogenic in prostate cancer [3,45]. Based on this observation *IL-6*/*STAT3* signaling inactivation was proposed as possible treatment for prostate cancer. However, targeting of this axis in patients has failed to provide therapeutic benefit. Moreover, it caused cancer progression to metastatic stages in a prostate cancer mouse model [3]. Overexpression of *STAT3* and its main regulator *IL6* is reported in the literature but *IL6* inhibitory effect in prostate cancer progression and failure of *STAT3*-*IL6* axis targeting as potential therapeutic target in patients were reported as well. Although *STAT3* and *IL6* have a definite impact on prostate cancer, because of the intrinsic molecular heterogeneity of prostate cancer, their role might be condition and stage dependent. Therefore, for the selection of the appropriate treatment strategy considering these two important factors a personalized medicine approach is required [46,47]. *STAT3* and almost all of the genes linked to *STAT3* in the network are involved in the JAK/STAT signaling pathway. *IL6ST*, which transduces the *IL6* signal to *STAT3*, is one of the most important genes in interaction with *STAT3*. Downregulation of *IL6* may be an important factor for the dysregulation of *STAT3* gene expression. Another important gene affecting *STAT3* is *IRF1*. *IRF1* encodes the interferon regulatory factor 1, and serves as an activator of interferon (IFN) alpha and beta transcription. *IRF1* also functions as a transcription activator of genes induced by IFN alpha, beta, and gamma. Further, *IRF1* has been shown to play roles in regulating apoptosis and tumor suppression [48]. Type-I Interferon (IFN-Alpha/Beta) promotes the DNA-binding activity of TFs including *STAT3* [49]. Thus, based on the metastatic state integrative regulatory network downregulation of *IRF1* may be the other reason for the inhibition of *STAT3*. Beyond these inhibitory factors our network introduces five overexpressed miRNAs (*miR-671-5p*, *miR-665*, *miR-663*, *miR-512-3p* and *miR-371-5p*) which have a suppressive effect on *STAT3* expression. Overexpression of *miR-663* is associated with the invasive phenotype of prostate cancer and was suggested as prognostic marker for high-risk prostate cancers. Overexpression of *miR-663* is associated with a downregulation of p21, which implies an impact of *miR-663* on G1/S checkpoint regulatory components [50]. Downregulation of miR-153, miR-155 and *miR-200c* was reported for high-grade prostatic intraepithelial neoplasia (HGPIN) and the initial stages of prostate cancer. These miRNAs were found to mediate overexpression of *STAT3* and *ZEB1*, which are key factors in high-grade prostatic intraepithelial neoplasia (HGPIN) and initial stages of prostate cancer [51]. However in the case of our network the synergistic effect of the above mentioned factors might cause a significant inhibition of *STAT3* in metastatic prostate tumors.

*JUN*, *JUNB*, *FOS*, *FOSB* and *ATF3* from the AP1 TF family are downregulated in the metastatic stage and are mediators of the TGF-β signaling pathway. Based on the results of our network analysis these genes may have a significant effect on the dysregulation of the TGF-β signaling pathway. Malfunctioning of this pathway is one of the factors promoting prostate cancer metastasis, which correlated well in our survival analysis. Low levels of *JUNB* in metastatic prostate cancer are associated with poor prognosis and advanced phases of prostate tumors. Two possible downstream effects were proposed for *JUNB*: the induction of *p16* associated cell cycle arrest and the suppression of *MMP2*, which is involved in the progression of prostate cancer. Moreover, downregulation of *JUNB* besides *PTEN* loss leads to metastatic prostate cancer. Therefore *JUNB* could serve as a biomarker for prostate cancer and for evaluating whether a given primary tumor has the potential to progress to metastatic phases [52–54].

*FOS* is common modulator of three key molecular signatures in the metastatic state. This gene is a family member of AP1. Direct interactions of *JUN* and *FOS* with *STAT3* have been observed in response to *IL6*. *JUN*/*STAT3* and *FOS*/*STAT3* complexes were detected on *IL6* response elements which lead to the transactivation of the *IL6* response elements through *STAT3* [55,56]. These observations indicate a close regulatory relationship of the key signatures in the constructed integrative regulatory network for metastatic prostate tumors.

*MYC* is a proto-oncogene and is overexpressed in a wide variety of tumors. Our TF analysis revealed that this gene is a mutual target of key molecular signatures in both primary and metastatic stages. In the primary stage it is overexpressed through *PGR*-based activation and in the metastatic stage it is suppressed by *STAT3* and *JUN*. However, due to weak correlation values these interactions were not considered in the TF regulatory networks. *MYC* is a regulator of cell growth and overexpression of *MYC* has been observed in the initial stages of prostate tumors [57,58]. Negative regulatory effects of *PGR* on *MYC* were reported for primary prostate tumors and in endometrial cancer [59,60]. In the metastatic prostate tumor *MYC* is downregulated due to *STAT3-*induced transcriptional suppression, however there are reports indicating that *STAT3* can also upregulate *MYC* in different kinds of cancer through *IL6* [61–66]. AP1 binding sites reside in the promoter region of *MYC* and binding of AP1 family members (*FOS* and *JUN*) resulted in the overexpression of *MYC* [67]. These links were previously highlighted in the epithelial to mesenchymal transition in prostate cancer and cancer in general [68].

In primary tumors four differentially expressed miRNAs including, *miR-139-5p*, *miR-501-3p*, *miR-630* and *miR-338-3p* target all the three key elements identified but they don’t have suppression effect on the key elements in the regulatory network of primary tumor. Interestingly, *miR-338-3p* commonly targets all the key elements in primary as well as in metastatic tumors. This miRNA is downregulated in both primary and metastatic prostate tumors as well as in several other tumors like gastric cancer, ovarian cancer, colorectal carcinoma and lung cancer [69,70].

Overall nine miRNAs in primary tumors (miR-20a*, *miR-32*, *miR-135a*, *miR-629*, *miR-19a*, *miR-19b*, *miR-175*, *miR-25* and *miR-548c-3p*) and six miRNAs in metastatic tumors (*miR-671-5p*, *miR-665*, *miR-663*, *miR-512-3p*, *miR-371-5p* and *miR-30d*) were identified in our networks suppressing key molecular elements. In order to evaluate the significance of these miRNAs in the initiation and progression of prostate cancer we further identified all the predicted targets of these miRNAs. In total, 1032 and 846 target genes were found for miRNA sets in primary and metastatic prostate tumors, respectively. Among these target genes, 474 and 421 genes were dysregulated in the prostate cancer. Gene set enrichment analysis was conducted on the dysregulated target genes in order to determine the affected pathways and biological processes. In particular, focal adhesion, ECM-receptor interaction, calcium signaling pathway, regulation of actin cytoskeleton and pathways in cancer are enriched for the target genes of miRNA set in primary tumor. In case of metastatic tumors, MAPK signaling pathway, focal adhesion, Jak-STAT signaling pathway, TGF-beta signaling pathway, melanoma, pancreatic cancer, colorectal cancer, prostate cancer and pathways in cancer are enriched pathways (S3–S5 Tables).

### Kaplan-Meier Survival analysis of patients to identify molecular signatures

**Fig 6:**
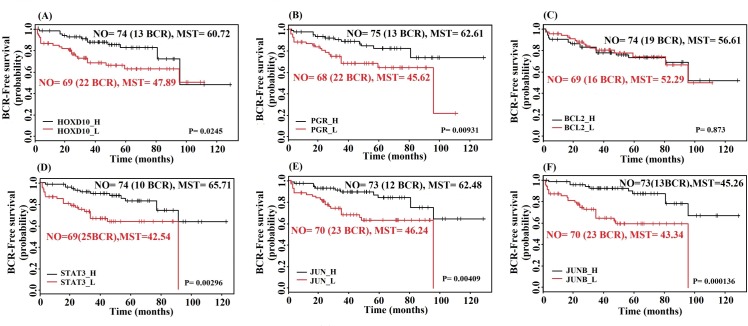
Kaplan-Meier survival curves of BCR-free survival probability for key molecular signatures. Plots 6A-6C show survival curves for key genes in primary prostate tumor and plots 6D-6F show survival plots for prostate cancer metastases. High expression of *STAT3* is associated with the highest mean survival time (MST = 65.71 months) and lowest BCR event (10 BCR events) among all of genes. NO: number of samples in the group, BCR: biochemical recurrence, MST: mean survival time.

For validating the importance of key network elements we conducted a Kaplan-Meier survival analysis using biochemical recurrence (BCR) data indicating tumor relapse in patients for selected molecular signatures identified through our integrative network analysis. Samples were grouped based on high and low expression values of each key molecular signature compared to their respective median expression values. We used BCR data as indicative factor of prostate tumors progression. Survival curves based on the key molecules (*HOXD10* and *PGR*) in primary prostate tumors shows significance differences between the high expression and low expression groups (P-value < 0.05). This means that patients with high expression of these molecules in primary prostate tumor have a higher chance to survive without BCR event in contrast to patients with low expression (Fig 6A and 6B). All of key genes in the metastatic state show significant difference for different expression groups. Among all key genes the highest and lowest mean survival times were observed for *STAT3* with 65.71 months (10 BCR events) and 42.54 months (25 BCR events) in the high and low expression groups, respectively (Fig 6D–6F).

## Discussion

The integrated TF-miRNA regulatory networks reveal some interesting information about molecular interactions in the primary and metastatic state of prostate cancer. We focused on positively as well as negatively correlating miRNA and mRNA pairs for the construction of miRNA regulatory network. Although the primary effect of miRNA-induced target regulation is the repression of target translation or its decay, we have also observed cases of positively correlating miRNA and mRNA expression profiles as in [33], which is in most of the cases a consequence of indirect target regulation, for instance, when a miRNA targets a transcriptional suppressor of another gene.

### Key molecules driving cancer progression

Overexpression of *EGR1*, *GDF15* and *MYC* in prostate cancer was previously reported in the literature [71–74]. Also the downregulation of *SNAI2*, *ITGA3*, *BCL2*, *PGR*, *IGFPB3* and *HOXD10* was previously reported for prostate cancer [8,36,75–78]. Based on the regulatory network analysis of primary prostate tumor, *HOXD10*, *BCL2* and *PGR* are 3 key elements in the network. Except for *BCL2* all these genes are TFs. Also in the survival analysis of *BCL2* high and low expression groups do not show any significant difference (Fig 6C). Our survival analysis and the regulatory pairs with significant correlation values indicate that *HOXD10* and *PGR* can be used as specific molecular signatures for the primary state in prostate cancer.

In the integrative network for metastatic prostate cancer *STAT3* may be the most important key regulator based on our network analysis, the literature and the results from the survival analysis (highest MST and least BCR events in the group with high *STAT3* expression). Moreover, many genes in the integrative regulatory network of the metastatic stage are members of the TGF-β receptor-signaling pathway. *SMAD4*, *SMAD9*, *TGFBR2*, *GDF15*, *JUN*, *FOS*, *JUNB*, *FOSB* and *ATF3* are involved in this pathway and strikingly, all of these genes were downregulated in the metastatic prostate tumor. Transforming growth factor β (TGF-β) is a multipotent cytokine that transduces the *TGFB1*, *TGFB2* and *TGFB3* signal from the cell surface to the cytoplasm, regulates a variety of cellular activities, such as cell proliferation, differentiation, and extracellular matrix (ECM) formation. Deprivation of TGF-β signaling was previously reported as prostate cancer metastases promoting factor [79]. For example, downregulation or loss of the *SMAD4* gene is associated with an aggressive phenotype of prostate cancer [80–83]. *JUN* and *JUNB* are the other key genes in the integrative regulatory network of the metastatic state. These genes are members of the activator protein 1 (AP1) TF family, which consists of a variety of dimers composed of members of the *FOS*, *JUN* and ATF families of proteins. AP1 converts extracellular signals into changes in the expression of specific target genes. This TF has been implicated in a large variety of biological processes such as cell differentiation, proliferation, apoptosis and oncogenic transformation [84]. Various studies have clarified the regulation of specific genes in response to TGF-β due to the functions of the AP1 family of TFs [85]. For the interactive transcriptional activity of TGF-beta, elements of the multimeric *SMAD3*/*SMAD4*/*JUN*/*FOS* complex are required [81]. *HOXB13* has been reported as overexpressed in castration- resistant prostate cancers [86]. It suppresses the expression of *p21* which is followed by the inhibition of AP1 signaling [86]. This may be the main reason for the downregulation of the AP1 TF in metastatic prostate cancer.

Most important, based on the integrative regulatory network and survival analysis, we confirm that *STAT3*, *JUN* and *JUNB* are the key molecules associated with the transition to metastatic prostate tumor. Our analysis also revealed that these TFs have a higher binding site frequency in targets that are part of our regulatory networks in comparison to random gene sets (S2 Fig).

Our results confirm the role of several key molecules in primary and metastatic prostate tumors. Among them, *HOXD10* and *PGR* are specific to primary tumor, while *STAT3*, *JUN* and *JUNB* are associated to metastatic tumor based on our integrative regulatory network and survival analysis. The results of this study suggest these molecules can be used as potent therapeutic targets, which however, requires further analyses. The approach used in the manuscript allows us to incorporate various data sources into an integrated network, analysis of network parameters in order to find key network elements and the validation of these findings using valuable clinical and pathological parameters. Using various data sources, we can substantiate the relationships between different molecular components to support our comprehension of how prostate cancer progresses. This approach may help unraveling regulatory mechanisms in other cancers as well.

## Supporting Information

S1 Fig Regulatory networks for (A) primary and (B) metastatic prostate cancer. The network for primary state contains 363 potential regulatory interactions between 41 differentially expressed miRNAs (DEMs) and 190 differentially expressed genes (DEGs) having absolute Pearson Correlation Coefficient (PCC) > 0.4. In case of metastatic regulatory network, we found in total 621 regulatory interactions between 79 miRNAs and 346 within the assigned PCC threshold. The edge color indicates the type of regulation (green for activation and red for repression) and the edge width is proportional to the absolute correlation coefficient for the expression values of the connected pair.
(TIF)

S2 Fig Binding sites distribution of key TFs in primary and metastatic tumor networks. The figure shows binding site distribution of key TFs in both primary (left side) and metastatic prostate (right side) cancer networks using the information available on TF binding sites in TRANSFAC database. The binding site frequency of key TFs on the promoter region of identified target genes is compared with that of random gene sets for each network. The comparison indicates that the binding site frequency of the key TFs for identified target genes in the networks is higher than the binding site frequency for random gene sets.
(TIF)

S1 Table TF and target gene regulatory pairs with corresponding PCC and correlation P-value for primary tumor. (DOCX)

S2 Table TF and target gene regulatory pairs with corresponding PCC and correlation p-value for metastatic tumor. (DOCX)

S3 Table Gene set enrichment analysis for DEGs connected to the miRNAs that target key molecular signatures shown in Table 1. (DOCX)

S4 Table Gene set enrichment analysis for DEGs connected to the miRNAs that target key molecular signatures shown in Table 1 for primary prostate cancer. (DOCX)

S5 Table Gene set enrichment analysis for DEGs connected to the miRNAs that target key molecular signatures shown in Table 1 for metastatic prostate cancer. (DOCX)
