# Supplementary figures and images for: MicroRNA and Transcription Factor Gene Regulatory Network Analysis Reveals Key Regulatory Elements Associated with Prostate Cancer Progression
Source: PLoS One. 2016 Dec 22;11(12):e0168760. doi: 10.1371/journal.pone.0168760 (PMC5179129; doi:10.1371/journal.pone.0168760)

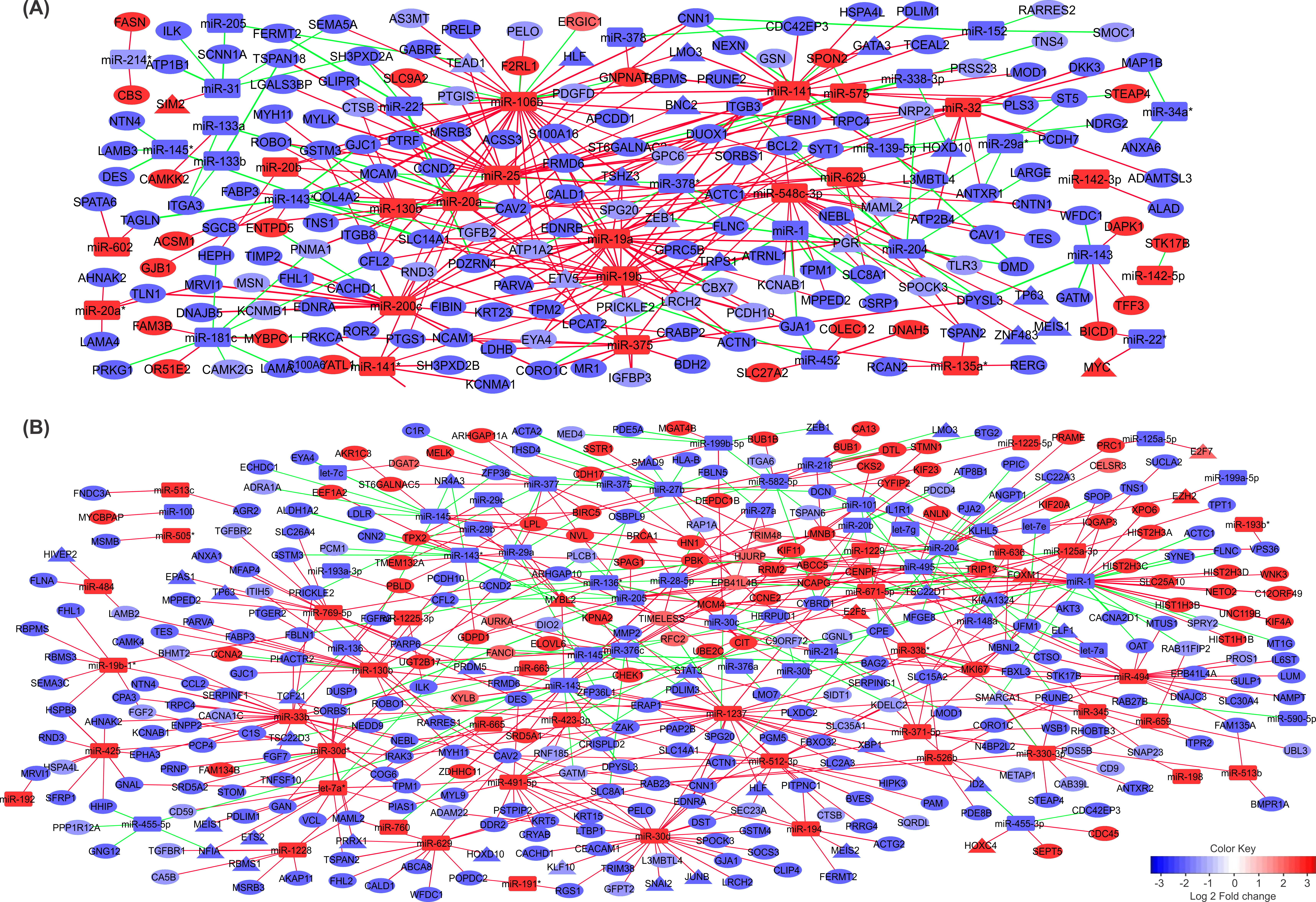

Supplement: S1 Fig — Regulatory networks for (A) primary and (B) metastatic prostate cancer. The network for primary state contains 363 potential regulatory interactions between 41 differentially expressed miRNAs (DEMs) and 190 differentially expressed genes (DEGs) having absolute Pearson Correlation Coefficient (PCC) > 0.4. In case of metastatic regulatory network, we found in total 621 regulatory interactions between 79 miRNAs and 346 within the assigned PCC threshold. The edge color indicates the type of regulation (green for activation and red for repression) and the edge width is proportional to the absolute correlation coefficient for the expression values of the connected pair. (TIF) [file pone.0168760.s001.tif]

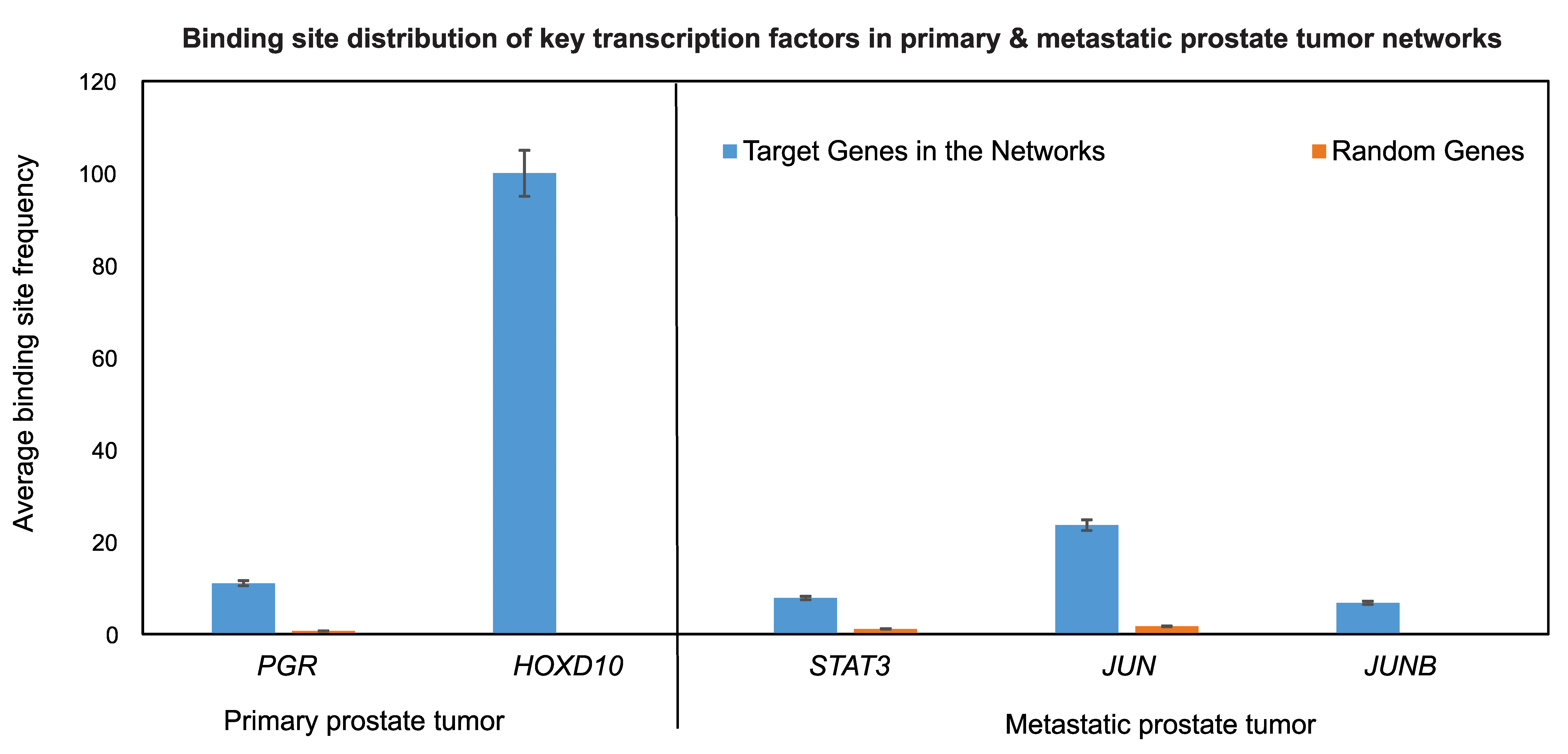

Supplement: S2 Fig — The figure shows binding site distribution of key TFs in both primary (left side) and metastatic prostate (right side) cancer networks using the information available on TF binding sites in TRANSFAC database. The binding site frequency of key TFs on the promoter region of identified target genes is compared with that of random gene sets for each network. The comparison indicates that the binding site frequency of the key TFs for identified target genes in the networks is higher than the binding site frequency for random gene sets. (TIF) [file pone.0168760.s002.tif]
