# Supplementary material for: MicroRNA and Transcription Factor Gene Regulatory Network Analysis Reveals Key Regulatory Elements Associated with Prostate Cancer Progression
Source: PLoS One. 2016 Dec 22;11(12):e0168760. doi: 10.1371/journal.pone.0168760 (PMC5179129; doi:10.1371/journal.pone.0168760)
Supplement: S1 Table — (DOCX) [file pone.0168760.s003.docx]

**Supplementary Table 1: TF and target gene regulatory pairs with corresponding PCC and correlation P-value for primary tumor**

| ***Significant TFs and target genes in Primary state*** | | | |
| --- | --- | --- | --- |
| **TF** | **Target Gene** | **PCC** | **p-values** |
| *TEAD1* | *ACTA2* | 0.711198 | 0 |
| *TEAD1* | *DES* | 0.691784 | 0 |
| *HOXD10* | *IGFBP3* | 0.436007 | 8.72E-05 |
| *SNAI2* | *ZEB1* | 0.529779 | 7.68E-11 |
| *ZEB1* | *ZEB1* | 1 | 0 |
| *SNAI2* | *ITGA3* | 0.403422 | 1.77E-06 |
| *MYC* | *MYC* | 1 | 0 |
| *TP63* | *TP63* | 1 | 0 |
| *TP63* | *FBXO32* | 0.470821 | 1.30E-05 |
| *EGR1* | *EGR1* | 1 | 0 |
| *EGR1* | *MAOB* | 0.44938 | 4.31E-05 |
| *EGR1* | *GDF15* | 0.416265 | 0.000233 |
| *EGR1* | *ACTA2* | 0.464197 | 1.90E-05 |
| *EGR1* | *SNAI2* | 0.469047 | 1.44E-05 |
| *EGR1* | *ZFP36* | 0.834566 | 0 |
| *EGR1* | *EGR1* | 1 | 0 |
| *GLI3* | *BCL2* | 0.404845 | 3.06E-06 |
| *PGR* | *BCL2* | 0.504036 | 8.39E-10 |
| *ZEB1* | *ACTA2* | 0.79428 | 0 |
| *PGR* | *ATP1B1* | 0.406748 | 1.43E-06 |
| *PGR* | *EDNRA* | 0.737396 | 0 |
| *BCL2* | *GLI3* | 0.424845 | 3.06E-06 |
|  |  |  |  |
