# Supplementary material for: MicroRNA and Transcription Factor Gene Regulatory Network Analysis Reveals Key Regulatory Elements Associated with Prostate Cancer Progression
Source: PLoS One. 2016 Dec 22;11(12):e0168760. doi: 10.1371/journal.pone.0168760 (PMC5179129; doi:10.1371/journal.pone.0168760)
Supplement: S2 Table — (DOCX) [file pone.0168760.s004.docx]

**Supplementary Table 2: TF and target gene regulatory pairs with corresponding PCC and correlation p-value for metastatic tumor**

| ***Significant TFs and target genes in Metastatic state*** | | | |
| --- | --- | --- | --- |
| ***TF*** | ***Target Gene*** | ***PCC*** | ***p-values*** |
| *EZH2* | *BRCA1* | 0.771735 | 0 |
| *BRIP1* | *BRCA1* | 0.874802 | 0 |
| *TOP2A* | *BRCA1* | 0.866768 | 0 |
| *AR* | *BRCA1* | 0.419039 | 9.45E-08 |
| *JUN* | *FOSB* | 0.716583 | 0 |
| *FOS* | *JUNB* | 0.818318 | 0 |
| *MYBL2* | *FOXM1* | 0.840799 | 0 |
| *ATF3* | *JUNB* | 0.678155 | 0 |
| *FOSB* | *JUNB* | 0.807221 | 0 |
| *FOS* | *STAT3* | 0.421319 | 7.92E-08 |
| *SMAD9* | *SMAD4* | 0.439674 | 1.81E-08 |
| *BRCA1* | *BRIP1* | 0.874802 | 0 |
| *BRCA1* | *EZH2* | 0.771735 | 0 |
| *AR* | *AR* | 1 | NA |
| *TSC22D1* | *SMAD4* | 0.624981 | 0 |
| *FOXM1* | *SMAD4* | -0.58708 | 2.89E-15 |
| *ATF3* | *ATF3* | 1 | NA |
| *EGR1* | *ATF3* | 0.684259 | 0 |
| *JUN* | *ATF3* | 0.782797 | 0 |
| *KLF4* | *ATF3* | 0.630302 | 0 |
| *ELF1* | *MEIS1* | 0.591553 | 1.55E-15 |
| *JUN* | *CTGF* | 0.48897 | 2.17E-10 |
| *STAT3* | *FOS* | 0.421319 | 7.92E-08 |
| *FOS* | *FOS* | 1 | NA |
| *STAT3* | *IRF1* | 0.487604 | 2.48E-10 |
| *IRF1* | *IRF1* | 1 | NA |
| *STAT3* | *MMP2* | 0.419169 | 9.36E-08 |
| *ETS2* | *MMP2* | 0.502821 | 5.49E-11 |
| *FOS* | *MMP2* | 0.400052 | 3.94E-07 |
| *JUNB* | *MMP2* | 0.404333 | 2.87E-07 |
| *STAT3* | *BIRC5* | -0.54672 | 4.57E-13 |
| *BHLHE40* | *BIRC5* | -0.4362 | 2.41E-08 |
| *KLF4* | *BIRC5* | -0.46102 | 2.91E-09 |
| *STAT3* | *IL6ST* | 0.638896 | 0 |
| *TP63* | *TP63* | 1 | NA |
| *KLF4* | *FOXM1* | -0.40457 | 2.82E-07 |
| *SNAI2* | *ZEB1* | 0.54739 | 4.22E-13 |
| *ZEB1* | *ZEB1* | 1 | NA |
| *STAT3* | *PROS1* | 0.436467 | 2.36E-08 |
| *PTTG1* | *STAT3* | -0.48515 | 3.14E-10 |
| *STAT3* | *GBP1* | 0.414631 | 1.33E-07 |
| *IRF1* | *GBP1* | 0.504004 | 4.87E-11 |
| *STAT3* | *SERPINA3* | 0.435839 | 2.48E-08 |
| *BHLHE40* | *BHLHE40* | 1 | NA |
| *JUN* | *BHLHE40* | 0.466229 | 1.82E-09 |
| *ATF3* | *SELE* | 0.525557 | 5.01E-12 |
| *JUN* | *SELE* | 0.529072 | 3.40E-12 |
| *TP63* | *KRT14* | 0.734288 | 0 |
| *TP63* | *FBXO32* | 0.43533 | 2.59E-08 |
| *EGR1* | *LDLR* | 0.436786 | 2.30E-08 |
| *KLF4* | *GDF15* | 0.48354 | 3.66E-10 |
| *ID2* | *EGR1* | 0.419751 | 8.95E-08 |
| *EGR1* | *ZFP36* | 0.790259 | 0 |
| *EGR1* | *KLF4* | 0.715468 | 0 |
| *KLF4* | *KLF4* | 1 | NA |
| *EGR1* | *EGR1* | 1 | NA |
| *EGR1* | *TGFBR2* | 0.426093 | 5.44E-08 |
| *EGR1* | *THBS1* | 0.434864 | 2.69E-08 |
| *IRF1* | *THBS1* | 0.564555 | 5.28E-14 |
| *JUN* | *JUN* | 1 | NA |
| *SMAD4* | *JUN* | 0.455578 | 4.69E-09 |
| *JUN* | *PTGS2* | 0.440136 | 1.74E-08 |
| *FOS* | *PTGS2* | 0.629696 | 0 |
| *FOS* | *ITGA5* | 0.452496 | 6.12E-09 |
| *NFIA* | *ITGA5* | 0.448258 | 8.81E-09 |
| *JUN* | *GADD45B* | 0.48384 | 3.56E-10 |
| *CEBPD* | *CCL2* | 0.645075 | 0 |
| *JUN* | *CYR61* | 0.686384 | 0 |
| *FOS* | *CYR61* | 0.725029 | 0 |
| *JUNB* | *CYR61* | 0.62327 | 0 |
| *FOS* | *PTGDS* | 0.418155 | 1.01E-07 |
| *NR4A1* | *JUN* | 0.728109 | 0 |
| *NR4A2* | *JUN* | 0.50602 | 3.96E-11 |
| *JUN* | *RGS2* | 0.515023 | 1.55E-11 |
| *E2F5* | *CDK1* | 0.491137 | 1.76E-10 |
| *ETS2* | *KIT* | 0.555952 | 1.52E-13 |
| *ETS2* | *BTG2* | 0.488766 | 2.21E-10 |
| *IRF1* | *CEACAM1* | 0.435036 | 2.65E-08 |
| *EPAS1* | *MEIS1* | 0.50191 | 6.02E-11 |
| *SMAD4* | *NDRG2* | -0.59615 | 8.88E-16 |
| *CDC6* | *FOXM1* | 0.711851 | 0 |
| *JUNB* | *IGFBP4* | 0.477343 | 6.57E-10 |
| *SMAD4* | *SEPP1* | 0.567225 | 3.80E-14 |
| *NFIB* | *ITGA6* | 0.595038 | 8.88E-16 |
| *EGR1* | *ZFP36* | 0.790259 | 0 |
| *EGR1* | *LDLR* | 0.436786 | 2.30E-08 |
| *EGR1* | *ATF3* | 0.684259 | 0 |
| *EGR1* | *KLF4* | 0.715468 | 0 |
| *ID2* | *EGR1* | 0.419751 | 8.95E-08 |
| *EGR1* | *TGFBR2* | 0.426093 | 5.44E-08 |
| *JUNB* | *IGFBP4* | 0.477343 | 6.57E-10 |
| *JUNB* | *MMP2* | 0.404333 | 2.87E-07 |
| *ATF3* | *LDLR* | 0.414997 | 1.29E-07 |
| *ATF3* | *SELE* | 0.525557 | 5.01E-12 |
| *IRF1* | *CEACAM1* | 0.435036 | 2.65E-08 |
| *IRF1* | *GBP1* | 0.504004 | 4.87E-11 |
| *STAT3* | *IL6ST* | 0.638896 | 0 |
| *STAT3* | *IRF1* | 0.487604 | 2.48E-10 |
| *STAT3* | *FOS* | 0.421319 | 7.92E-08 |
| *STAT3* | *MMP2* | 0.419169 | 9.36E-08 |
| *STAT3* | *PROS1* | 0.436467 | 2.36E-08 |
| *PTTG1* | *STAT3* | -0.48515 | 3.14E-10 |
| *STAT3* | *BIRC5* | -0.54672 | 4.57E-13 |
| *EPAS1* | *MEIS1* | 0.50191 | 6.02E-11 |
| *STAT3* | *SOCS3* | 0.458825 | 3.53E-09 |
| *ELF1* | *MEIS1* | 0.591553 | 1.55E-15 |
| *IRF1* | *HLA-B* | 0.45689 | 4.18E-09 |
| *JUNB* | *JUN* | 0.642357 | 0 |
