# Supplementary material for: MicroRNA and Transcription Factor Gene Regulatory Network Analysis Reveals Key Regulatory Elements Associated with Prostate Cancer Progression
Source: PLoS One. 2016 Dec 22;11(12):e0168760. doi: 10.1371/journal.pone.0168760 (PMC5179129; doi:10.1371/journal.pone.0168760)
Supplement: S3 Table — (DOCX) [file pone.0168760.s005.docx]

**Supplementary Table 3: Gene set enrichment analysis for DEGs connected to the miRNAs that target key molecular signatures shown in table 1.**

| **Gene Set Enrichment Analysis for KEGG pathways** | | | | |
| --- | --- | --- | --- | --- |
|  | Enriched KEGG pathway | p-value | Set.size |  |
| **Primary tumors** | hsa04510 Focal adhesion | 2.72E-08 | 25 | **Overexpressed KEGG pathways** |
|  | hsa04810 Regulation of actin cytoskeleton | 4.24E-07 | 10 |  |
|  | hsa04020 Calcium signaling pathway | 1.21E-05 | 10 |  |
|  | hsa04512 ECM-receptor interaction | 0.010614 | 10 |  |
|  | hsa05200 Pathways in cancer | 0.036869 | 24 |  |
| **Metastatic Tumor** | hsa04510 Focal adhesion | 0.000151 | 20 | **Suppressed KEGG pathways** |
|  | hsa04810 Regulation of actin cytoskeleton | 0.000453 | 14 |  |
|  | hsa04010 MAPK signaling pathway | 0.000614 | 10 |  |
|  | hsa04350 TGF-beta signaling pathway | 0.010921 | 12 |  |
|  | hsa04630 Jak-STAT signaling pathway | 0.024187 | 11 |  |
|  | hsa05218 Melanoma | 0.0034 | 10 |  |
|  | hsa05212 Pancreatic cancer | 0.003948 | 11 |  |
|  | hsa05210 Colorectal cancer | 0.005391 | 10 |  |
|  | hsa05200 Pathways in cancer | 0.006362 | 34 |  |
|  | hsa05215 Prostate cancer | 0.007021 | 12 |  |
|  | hsa00230 Purine metabolism | 4.54E-05 | 10 | **Overexpressed KEGG pathways** |
