# Supplementary material for: MicroRNA and Transcription Factor Gene Regulatory Network Analysis Reveals Key Regulatory Elements Associated with Prostate Cancer Progression
Source: PLoS One. 2016 Dec 22;11(12):e0168760. doi: 10.1371/journal.pone.0168760 (PMC5179129; doi:10.1371/journal.pone.0168760)
Supplement: S4 Table — (DOCX) [file pone.0168760.s006.docx]

**Supplementary Table 4: Gene set enrichment analysis for DEGs connected to the miRNAs that target key molecular signatures shown in table 1 for primary prostate cancer.**

| **Gene Set Enrichment Analysis for GO.BP term** | | | | |
| --- | --- | --- | --- | --- |
|  | **GO.BP term** | **p-value** | **Set.size** |  |
| **Primary tumors** | GO:0008219 cell death | 2.12E-08 | 79 | **Suppressed GO.BP terms** |
|  | GO:0016265 death | 2.12E-08 | 79 |  |
|  | GO:0006915 apoptotic process | 4.51E-07 | 75 |  |
|  | GO:0001817 regulation of cytokine production | 6.15E-07 | 25 |  |
|  | GO:0010941 regulation of cell death | 4.20E-06 | 65 |  |
|  | GO:0031328 positive regulation of cellular biosynthetic process | 4.63E-06 | 63 |  |
|  | GO:0043066 negative regulation of apoptotic process | 1.41E-05 | 42 |  |
|  | GO:0022409 positive regulation of cell-cell adhesion | 1.59E-05 | 14 |  |
|  | GO:0042981 regulation of apoptotic process | 1.90E-05 | 62 |  |
|  | GO:0010558 negative regulation of macromolecule biosynthetic process | 2.08E-05 | 57 |  |
|  | GO:0043409 negative regulation of MAPK cascade | 3.87E-05 | 11 |  |
|  | GO:0007049 cell cycle | 5.36E-05 | 68 |  |
|  | GO:0008283 cell proliferation | 5.54E-05 | 97 |  |
|  | GO:0000165 MAPK cascade | 8.94E-05 | 40 |  |
|  | GO:0043281 regulation of cysteine-type endopeptidase activity involved in apoptotic process | 8.98E-05 | 12 |  |
|  | GO:0060070 canonical Wnt signaling pathway | 0.000115 | 17 |  |
|  | GO:0030155 regulation of cell adhesion | 0.000187 | 39 |  |
|  | GO:0009968 negative regulation of signal transduction | 0.000193 | 57 |  |
|  | GO:0031098 stress-activated protein kinase signaling cascade | 0.000202 | 10 |  |
|  | GO:0051403 stress-activated MAPK cascade | 0.000202 | 10 |  |
|  | GO:0051726 regulation of cell cycle | 0.000544 | 46 |  |
|  | GO:0070371 ERK1 and ERK2 cascade | 0.000606 | 17 |  |
|  | GO:0070372 regulation of ERK1 and ERK2 cascade | 0.000606 | 17 |  |
|  | GO:0045765 regulation of angiogenesis | 0.000768 | 17 |  |
|  | GO:0097285 cell-type specific apoptotic process | 0.000768 | 22 |  |
|  | GO:0043408 regulation of MAPK cascade | 0.000863 | 39 |  |
|  | GO:0051234 establishment of localization | 0.000875 | 141 |  |
|  | GO:0031100 organ regeneration | 0.000888 | 10 |  |
|  | GO:0018193 peptidyl-amino acid modification | 0.000914 | 44 |  |
|  | GO:0043406 positive regulation of MAP kinase activity | 0.000959 | 12 |  |
|  | GO:0019222 regulation of metabolic process | 0.001008 | 200 |  |
|  | GO:0051248 negative regulation of protein metabolic process | 0.002819 | 47 |  |
|  | GO:0001525 angiogenesis | 0.002873 | 32 |  |
|  | GO:0070848 response to growth factor | 0.004543 | 46 |  |
|  | GO:0045786 negative regulation of cell cycle | 0.00958 | 20 |  |
|  | GO:0043603 cellular amide metabolic process | 0.009848 | 23 |  |
|  | GO:0019220 regulation of phosphate metabolic process | 0.009895 | 73 |  |
|  | GO:0051174 regulation of phosphorus metabolic process | 0.009895 | 73 |  |
|  | GO:0002440 production of molecular mediator of immune response | 0.009909 | 12 |  |
|  | GO:0044257 cellular protein catabolic process | 0.009955 | 15 |  |
|  | GO:0051603 proteolysis involved in cellular protein catabolic process | 0.009955 | 15 |  |
|  | GO:0006518 peptide metabolic process | 0.010369 | 16 |  |
|  | GO:0031347 regulation of defense response | 0.010432 | 32 |  |
|  | GO:0010942 positive regulation of cell death | 0.013099 | 30 |  |
|  | GO:0043065 positive regulation of apoptotic process | 0.013099 | 30 |  |
|  | GO:0043068 positive regulation of programmed cell death | 0.013099 | 30 |  |
|  | GO:0051090 regulation of sequence-specific DNA binding transcription factor activity | 0.022594 | 20 |  |
|  | GO:0016055 Wnt signaling pathway | 0.023663 | 24 |  |
|  | GO:0071345 cellular response to cytokine stimulus | 0.026141 | 31 |  |
|  | GO:0071902 positive regulation of protein serine/threonine kinase activity | 0.02644 | 16 |  |
|  | GO:0060485 mesenchyme development | 0.031448 | 17 |  |
|  | GO:0060828 regulation of canonical Wnt signaling pathway | 0.032446 | 11 |  |
|  | GO:0034599 cellular response to oxidative stress | 0.036898 | 14 |  |
|  | GO:0030029 actin filament-based process | 2.03E-13 | 28 | **Overexpressed GO.BP terms** |
|  | GO:0030048 actin filament-based movement | 4.23E-13 | 10 |  |
|  | GO:0032990 cell part morphogenesis | 9.12E-12 | 37 |  |
|  | GO:0007265 Ras protein signal transduction | 0.000651 | 11 |  |
|  | GO:0051493 regulation of cytoskeleton organization | 0.000749 | 14 |  |
|  | GO:0072507 divalent inorganic cation homeostasis | 0.000777 | 22 |  |
|  | GO:0045927 positive regulation of growth | 0.001832 | 11 |  |
|  | GO:1902589 single-organism organelle organization | 0.001937 | 65 |  |
|  | GO:0072503 cellular divalent inorganic cation homeostasis | 0.001944 | 21 |  |
|  | GO:0007264 small GTPase mediated signal transduction | 0.005235 | 25 |  |
|  | GO:0010720 positive regulation of cell development | 0.005572 | 27 |  |
|  | GO:0007626 locomotory behavior | 0.005753 | 15 |  |
