# Supplementary material for: MicroRNA and Transcription Factor Gene Regulatory Network Analysis Reveals Key Regulatory Elements Associated with Prostate Cancer Progression
Source: PLoS One. 2016 Dec 22;11(12):e0168760. doi: 10.1371/journal.pone.0168760 (PMC5179129; doi:10.1371/journal.pone.0168760)
Supplement: S5 Table — (DOCX) [file pone.0168760.s007.docx]

**Supplementary Table 5:** **Gene set enrichment analysis for DEGs connected to the miRNAs that target key molecular signatures shown in table 1 for metastatic prostate cancer.**

| **Gene Set Enrichment Analysis for GO.BP terms** | | | | |
| --- | --- | --- | --- | --- |
|  | **GO.BP term** | **p-value** | **Set.size** |  |
| **Metastatic tumors** | GO:0030198 extracellular matrix organization | 2.41E-09 | 33 | **Suppressed GO.BP terms** |
|  | GO:0030512 negative regulation of transforming growth factor beta receptor signaling pathway | 2.41E-09 | 33 |  |
|  | GO:0034329 cell junction assembly | 5.70E-07 | 21 |  |
|  | GO:0034330 cell junction organization | 5.70E-07 | 21 |  |
|  | GO:0010810 regulation of cell-substrate adhesion | 5.96E-07 | 14 |  |
|  | GO:0070371 ERK1 and ERK2 cascade | 6.82E-07 | 10 |  |
|  | GO:0070372 regulation of ERK1 and ERK2 cascade | 6.82E-07 | 10 |  |
|  | GO:0001525 angiogenesis | 1.39E-06 | 20 |  |
|  | GO:0031589 cell-substrate adhesion | 2.56E-06 | 24 |  |
|  | GO:0071559 response to transforming growth factor beta | 2.64E-06 | 22 |  |
|  | GO:0043405 regulation of MAP kinase activity | 1.37E-05 | 17 |  |
|  | GO:0007229 integrin-mediated signaling pathway | 2.69E-05 | 11 |  |
|  | GO:0006874 cellular calcium ion homeostasis | 4.00E-05 | 11 |  |
|  | GO:0055074 calcium ion homeostasis | 4.00E-05 | 11 |  |
|  | GO:0038127 ERBB signaling pathway | 5.01E-05 | 10 |  |
|  | GO:0030154 cell differentiation | 5.44E-05 | 128 |  |
|  | GO:0071363 cellular response to growth factor stimulus | 6.59E-05 | 36 |  |
|  | GO:0043410 positive regulation of MAPK cascade | 7.01E-05 | 19 |  |
|  | GO:0007155 cell adhesion | 9.26E-05 | 80 |  |
|  | GO:0043408 regulation of MAPK cascade | 0.000112 | 30 |  |
|  | GO:0007259 JAK-STAT cascade | 0.000122 | 10 |  |
|  | GO:0097696 STAT cascade | 0.000364 | 10 |  |
|  | GO:0007179 transforming growth factor beta receptor signaling pathway | 0.000364 | 24 |  |
|  | GO:0045597 positive regulation of cell differentiation | 0.000366 | 36 |  |
|  | GO:0050795 regulation of behavior | 0.000369 | 11 |  |
|  | GO:0043067 regulation of programmed cell death | 0.000419 | 45 |  |
|  | GO:0008219 cell death | 0.000442 | 59 |  |
|  | GO:0016265 death | 0.000442 | 59 |  |
|  | GO:0042509 regulation of tyrosine phosphorylation of STAT protein | 0.000456 | 43 |  |
|  | GO:0060397 JAK-STAT cascade involved in growth hormone signaling pathway | 0.000474 | 20 |  |
|  | GO:0007160 cell-matrix adhesion | 0.000603 | 14 |  |
|  | GO:0000165 MAPK cascade | 0.000766 | 32 |  |
|  | GO:0071604 transforming growth factor beta production | 0.000887 | 27 |  |
|  | GO:0097285 cell-type specific apoptotic process | 0.001192 | 15 |  |
|  | GO:0051270 regulation of cellular component movement | 0.001341 | 40 |  |
|  | GO:0012501 programmed cell death | 0.001391 | 56 |  |
|  | GO:0007260 tyrosine phosphorylation of STAT protein | 0.001444 | 31 |  |
|  | GO:0043069 negative regulation of programmed cell death | 0.001519 | 32 |  |
|  | GO:0098609 cell-cell adhesion | 0.001533 | 43 |  |
|  | GO:0019058 viral life cycle | 0.002759 | 11 |  |
|  | GO:0031347 regulation of defense response | 0.002798 | 26 |  |
|  | GO:0009101 glycoprotein biosynthetic process | 0.002801 | 25 |  |
|  | GO:0060070 canonical Wnt signaling pathway | 0.003936 | 10 |  |
|  | GO:0051253 negative regulation of RNA metabolic process | 0.003943 | 35 |  |
|  | GO:0034097 response to cytokine | 0.007059 | 33 |  |
|  | GO:0048598 embryonic morphogenesis | 0.007077 | 21 |  |
|  | GO:0070838 divalent metal ion transport | 0.007287 | 16 |  |
|  | GO:0072511 divalent inorganic cation transport | 0.007287 | 16 |  |
|  | GO:0048608 reproductive structure development | 0.007402 | 19 |  |
|  | GO:0061458 reproductive system development | 0.007402 | 19 |  |
|  | GO:0007186 G-protein coupled receptor signaling pathway | 0.007449 | 21 |  |
|  | GO:0046427 positive regulation of JAK-STAT cascade | 0.017508 | 30 |  |
|  | GO:0019221 cytokine-mediated signaling pathway | 0.023462 | 19 |  |
|  | GO:0060326 cell chemotaxis | 0.023478 | 12 |  |
|  | GO:0022402 cell cycle process | 6.79E-21 | 39 | **Overexpressed GO.BP terms** |
|  | GO:0000278 mitotic cell cycle | 1.76E-13 | 31 |  |
|  | GO:0007049 cell cycle | 4.41E-13 | 59 |  |
|  | GO:0051276 chromosome organization | 6.54E-09 | 14 |  |
|  | GO:0000280 nuclear division | 2.08E-08 | 16 |  |
|  | GO:0000082 G1/S transition of mitotic cell cycle | 2.72E-06 | 10 |  |
|  | GO:0044843 cell cycle G1/S phase transition | 2.72E-06 | 10 |  |
|  | GO:0051301 cell division | 5.92E-05 | 22 |  |
|  | GO:0006091 generation of precursor metabolites and energy | 0.000333 | 11 |  |
|  | GO:0000226 microtubule cytoskeleton organization | 0.000358 | 15 |  |
|  | GO:0007067 mitotic nuclear division | 0.000442 | 12 |  |
|  | GO:0007267 cell-cell signaling | 0.000703 | 30 |  |
